# Associations of healthy lifestyles and physical education enjoyment with physical fitness among Chinese medical students: a cross-sectional study

**DOI:** 10.3389/fpubh.2026.1894015

**Published:** 2026-08-26

**Authors:** Xuechen Mao, Yun Zhang

**Affiliations:** 1 Department of Physical Education, Nanjing University of Chinese Medicine, Nanjing, China; 2 Department of Child and Adolescent Psychiatry, The Second People's Hospital of Jiangning District, Nanjing, China

**Keywords:** Chinese medical students, healthy lifestyle, physical education enjoyment, physical fitness, subjective initiative

## Abstract

**Background:**

Physical fitness is a key indicator for assessing individuals’ current health status and predicting future health outcomes. However, relatively little attention has been paid to the physical fitness of medical students, who will serve as the main workforce in the healthcare industry in the future. This cross-sectional study aimed to explore the current status of physical fitness and to examine the associations of healthy lifestyles and physical education (PE) enjoyment with physical fitness among Chinese medical students.

**Methods:**

A total of 12,840 students from three medical universities in Jiangsu Province were enrolled. Validated questionnaires were used to assess healthy lifestyles and PE enjoyment, while physical fitness indicators were measured according to national standards. Multiple regression was used to determine the relationship between independent variables (participants’ basic information, health-related lifestyle and enjoyment of PE) and dependent variables (participants’ physical fitness performance).

**Results:**

The results showed that medical students exhibited a moderate level of physical fitness (70.85 ± 0.33), which was influenced by demographic factors including gender, major type, academic system, grade and monthly living expenses. Moreover, medical students constituted a highly sedentary population, with daily screen time of 6.56 ± 3.20 h and daily non-screen sedentary time of 4.56 ± 3.10 h. Their physical fitness was positively correlated with exercise frequency, sleep duration, non-screen sedentary time, and PE enjoyment, and negatively correlated with screen time and smoking behavior. Furthermore, after incorporating PE enjoyment, the negative correlation between smoking and physical health was not significant (*p* > 0.05).

**Conclusion:**

Although relatively uncontrollable factors (e.g., gender, major, academic system, grade, and monthly living expenses) show significant correlations with physical fitness, relatively self-controllable factors (healthy lifestyle habits) and interactions with the external environment (PE enjoyment) also exhibit strong associations with physical fitness. The results suggest that fostering healthy lifestyle behaviors through individuals’ internal motivation and improving the quality of physical education classes may be of great significance for medical students to achieve superior physical fitness levels.

## 1 Introduction

Physical fitness is a comprehensive indicator reflecting cardiorespiratory function, body composition, muscular strength and endurance, flexibility, balance, speed, and explosive power (1, 2). It serves as a key marker of an individual’s current and future health status (3, 4). Better physical fitness not only enhances bodily functions but also improves psychological well-being, promotes social interactions, and boosts cognitive and academic performance (2, 5). Given the beneficial effects of physical fitness on health, many countries have issued 24-h movement guidelines for people of different age groups to promote physical health (6–8), and established comprehensive physical fitness monitoring systems, such as the Chinese National Student Physical Fitness Test (4). Nevertheless, due to the rising prevalence of obesity caused by unhealthy lifestyles, the physical fitness of populations worldwide, especially among students, has continued to decline (9–11).

A large number of studies have demonstrated that unhealthy lifestyles, including physical inactivity, prolonged sedentary behavior, and insufficient sleep, are critical risk factors for poor physical fitness (12–15). University students, who spend considerable time in classes and on computers, are particularly vulnerable to developing sedentary behaviors (3, 16). Although sufficient physical activity can effectively reduce sedentary behavior, improve sleep, and alleviate the harmful effects of prolonged sitting (17), most students fail to meet the daily recommended physical activity levels (18). In particular, physical activity frequency decreased during the COVID-19 pandemic, and sedentary behavior among students increased in the post-pandemic period, leading to a continuous decline in physical fitness (19), which has even fallen below that of middle school students (20).

Fortunately, sleep, physical activity, and sedentary behavior are modifiable behaviors among university students (14). Numerous studies have found that physical education (PE) can increase physical activity intensity, improve health literacy, and prevent diseases (21). Therefore, the Ministry of Education of China, universities, and PE teachers are attempting to promote healthy lifestyles among university students and reverse the downward trend in physical fitness through effective interventions such as increasing PE courses and diversifying PE content. However, traditional physical education courses focus solely on the teaching of motor skills with relatively monotonous content, resulting in low student satisfaction with physical education. Additionally, previous studies focusing on PE courses have mostly centered on the design of learning objectives, course content plans, and appropriate course workloads, with relatively little attention paid to students’ enjoyment during PE classes. Yet this is precisely the key to motivating students to participate actively in PE (22, 23). Therefore, at present, university students’ participation in PE is relatively low, and the exercise intensity in PE classes does not meet the required standards, which greatly weakens the intervention effect of PE on students’ physical fitness (24). Studies have shown that students’ enjoyment in PE can improve their exercise participation (25, 26), thereby increasing exercise intensity (27) and reducing obesity rates (16, 28). Other research has found that personalized care and encouragement from PE teachers can enhance students’ enjoyment in PE (29, 30), thus promoting autonomous exercise behavior (31, 32). Therefore, many scholars have suggested that research should be conducted for specific populations and targeted PE curricula should be developed (33–35).

Among all university students, medical students represent a special group of future healthcare providers. Their physical and mental health not only affects their academic performance and quality of life but also determines their professional competence and long-term service capacity in clinical practice (36, 37). Therefore, the physical fitness of medical students is particularly critical. However, research has documented a declining trend in physical fitness among Chinese medical students (37). Medical education in China typically follows a 5-year undergraduate program, with 8-year combined bachelor-master and 9-year combined bachelor-doctor programs also available. Students must not only master an extensive body of medical knowledge within a limited timeframe but also complete standardized residency training and clinical clerkships in hospitals and pass corresponding assessments. Owing to the unique demands of their profession, they face heavy academic burdens, high psychological stress, and prolonged sedentary behavior, with little spare time for physical activity, which making them a high-risk group for unhealthy lifestyles (38). Although some studies have suggested that medical students possess better health literacy compared with students in other majors (39–41), medical students are burdened with heavy curricula, high academic pressure, prolonged sedentary time and insufficient physical activity, resulting in poorer overall physical fitness than non-medical students (42, 43). Noteworthy, physical education classes not only promote physical fitness but also enhance working memory (44), improve academic achievement (36, 45, 46), foster social skills, and develop problem-solving abilities (47). In doing so, PE contributes to holistic physical and mental health, as well as comprehensive cognitive and personality development. Thus, PE is of paramount importance for medical students. However, to date, there are relatively few studies on the physical fitness status of Chinese medical students, and even fewer targeted recommendations for PE interventions.

In summary, previous research has focused on either lifestyle factors or PE enjoyment separately, lacking an integrated analysis. Such an integrated analysis could provide valuable evidence and recommendations for the Ministry of Education, universities, and physical education teachers to implement physical education curriculum reform. Moreover, urgent attention and effective measures are needed to address and reverse the declining physical fitness trend among Chinese medical students. Therefore, this cross-sectional study was conducted to investigate the current status of physical fitness among Chinese medical students and to examine the associations of healthy lifestyles and PE enjoyment with physical fitness. The findings are expected to offer evidence-based suggestions for optimizing PE curricula and implementing targeted healthy lifestyle education, thereby improving the physical fitness of medical students and supporting the implementation of China’s Sports Power initiative.

## 2 Materials and methods

### 2.1 Study design and procedure

This study employed a non-experimental, cross-sectional design. It aimed to explore the current status of physical fitness among contemporary Chinese medical students, and comprehensively and ecologically validly reveal the associations between PE enjoyment, health-related lifestyles, and their physical fitness. To comprehensively assess students’ physical health status and promote their physical fitness, universities in China annually arrange physical education teachers to conduct physical fitness tests among enrolled students. To encourage greater student participation in the present study, researchers distributed electronic brochures to each administrative class in medical universities and promised to safeguard the privacy of all participants.

After understanding the general purpose of the study and signing the informed consent form, participants came to the playground in batches. They first completed all items of the physical fitness test in sequence (See Section 2.3 for details), and then received a Questionnaire Star link^1^ to fill out the questionnaire. Upon completion of all tasks, they registered with the instructor before leaving. The entire procedure was finished within one and a half hours. The instruments and test methods, test content, and scoring standards used in the physical fitness test are strictly in accordance with the content and requirements stipulated in the National Student physical fitness standard issued by the Ministry of Education of the People’s Republic of China (see Table 1 and Section 2.3.1 for details). Data were recorded using standardized electronic devices for physical fitness testing mandated by the state (Smart Sports Handheld Terminal). Two PE teachers were assigned to record data at each testing site, and the mean value of the two recordings was taken as the final score to minimize errors. If a large discrepancy was observed between the records of the two teachers, a second test and recording session was rescheduled. All participating PE teachers had received unified training and assessment from the provincial education bureau to ensure the accuracy, as well as professional safety and first-aid training with certified qualifications to enforce risk control. Furthermore, students are given systematic training and guidance on the physical fitness test in PE classes in advance. Standard warm-up and post-test stretching sessions are arranged, while medical personnel and rescue facilities are present at all test sites. Centralized training was provided for data collectors from September 2024 to February 2025, during which the research objectives and data collection methods were explained. Data collection was then conducted from March to September 2025. The study was approved by the Ethics Committee of the Affiliated Hospital of Nanjing University of Chinese Medicine (protocol No. SQ2024051). All participants signed an informed consent form as part of the inclusion criteria.

**Table 1:** Physical fitness tests and evaluation indications.

Single index	Weight (%)	
	Male	Female
Body mass index	15	15
Vital capacity	15	15
50-m sprint	20	20
Sit-and-reach	10	10
Standing long jump	10	10
Pull-ups	10	/
Bent-leg sit-ups	/	10
1,000-m run	20	/
800-m run	/	20
Total	100	100

### 2.2 Participants

There exists uneven regional development across China, with corresponding geographical variations in physical fitness levels (48). Different from other regions, Jiangsu Province has a moderate economic status nationwide and is home to a large number of medical universities, enabling the acquisition of substantial data. In addition, all researchers are from Jiangsu, which facilitates unified personnel deployment, centralized test arrangement, and error reduction. Therefore, convenience sampling was adopted in this study, and participants were selected from three representative medical universities with a total of four campuses in Jiangsu, including one 211 Project university, one Double First-Class university, and one ordinary undergraduate university.

There are two definitions of medical students. In a narrow sense, medical students refer to those majoring in clinical medicine, in a broad sense, they include students majoring in medicine-related disciplines. The students of the medical university investigated in this study fall into the broad definition, consisting of clinical majors (e.g., internal medicine, surgery, obstetrics and gynecology, etc.) and non-clinical majors (e.g., pharmacy, basic medical sciences, forensic medicine, etc.).

Inclusion criteria: (1) full-time Chinese undergraduate students, (2) informed consent and signing an informed consent form. Exclusion criteria: (1) Participants who failed to complete either the physical fitness test or the questionnaire, (2) There were obvious regularity or abnormal values in the content of the physical fitness test or the questionnaire. Missing data occurred when participants were unable to complete all physical fitness tests or questionnaires during data collection for various reasons (e.g., injury, suspension of study), or when some data contained obvious errors (e.g., total sedentary time plus sleep time exceeding 24 h). Data from the above situations were excluded and not included in the final statistical analysis. A total of 13,562 participants were recruited in this study, and 12,840 were finally included in the analysis, with a response rate of 94.68%.

### 2.3 Study measures

#### 2.3.1 Physical fitness tests

Data collection instructors strictly adhere to the physical fitness testing standards set by the Chinese government, ensuring a nationally unified testing procedure and measurement criteria, which guarantees the quality of the data. The test items for males and females are not identical, and their respective percentage weightings are shown in Table 1. The participants were required to warm up before taking the physical fitness tests. Based on the total scores, participants were classified into four performance levels: Excellent (90.0 or higher), Good (80.0 to 89.9), Pass (60.0 to 79.9), and Fail (less than 60.0).

##### 2.3.1.1 Test of body mass index (BMI)

BMI is employed to evaluate body symmetry and proportionality. The formula used for calculating BMI is weight (kg)/height (m)^2^. During the test, participants were required to remove their shoes, keep their trunk and head stationary, and place the top of the head directly below the probe of the instrument.

##### 2.3.1.2 Vital capacity

Vital capacity is defined as the total volume of air that can be displaced from the lungs by maximal expiratory effort, thus it evaluated pulmonary volume and distensibility, serving as a key indicator for assessing respiratory system function. Students performed the test using an electronic spirometer with a dry plastic mouthpiece. During the measurement, each student took a deep breath and then exhaled slowly into the mouthpiece until no more air could be expelled. After the exhalation, the final value displayed on the LCD screen represented the vital capacity in milliliters. Each student completed two trials with a 15-s interval between them, and the maximum value was recorded as the official test result. The results were expressed in milliliters with no decimal places retained.

##### 2.3.1.3 50-m sprint

The 50-meter sprint assesses speed ability through high-intensity running over a short distance. When the investigator said “go,” the participants began the 50-m run. They finished the run as fast as they could. The time recoding was measured in seconds, and each participant was permitted two attempts, and the better performance was recorded.

##### 2.3.1.4 Sit-and-reach

The sit-and-reach test is a measurement item used to reflect the flexibility of the human body. During the test, participants were required to keep their legs straight and the soles of their feet firmly against the test board. When bending forward, they pushed the slider at a constant speed, avoiding knee flexion or sudden exertion to accurately reflect the flexibility of the lower back and the posterior thigh muscles. Each student was permitted two attempts, and the furthest distance was recorded in centimeters.

##### 2.3.1.5 Standing long jump

Standing long jump measures the explosive force of the lower limb muscles during forward jumping and is an indicator for evaluating lower limb explosive force. During the test, students stood with their feet naturally apart behind the take-off line, with their toes not touching the line. They jumped forward simultaneously from a stationary position with both feet, and a hop or consecutive jumps were not permitted. The vertical distance from the rear edge of the take-off line to the rear edge of the nearest landing point was measured. Each student was allowed three trial jumps, and the best performance was recorded as the final result, expressed in centimeters with no decimal places retained.

##### 2.3.1.6 Pull-ups

Pull-ups mainly reflect the development level of relative strength and endurance of the upper limb muscles. During the test, the subject stood naturally facing the horizontal bar, grasped the bar with a pronated grip at shoulder width, and maintained the body in a straight-arm hanging position. Once body oscillation ceased, participants pulled their body upward using simultaneous force from both arms until their mandible rose above the upper edge of the horizontal bar. Returning to the straight-arm hanging position constituted one full repetition. The interval between consecutive pull-ups was limited to no more than 10 s. Excessive body swinging or the use of any auxiliary movements to assist elevation was prohibited during the exercise. The total number of valid pull-ups was recorded.

##### 2.3.1.7 Bent-leg sit-ups

Sit-ups mainly measure the strength and endurance of the abdominal muscles. During the test, the subject lay supine on a horizontally placed mat with legs slightly apart and knees bent at 90 degrees. The fingers were interlocked and placed behind the head, and a partner held down the subject’s feet to fix the lower limbs. The tester started the stopwatch at the same time as giving the start command. The subject performed sit-ups quickly until both elbows touched or exceeded the knees, then returned to the supine position with both shoulder blades touching the mat, this sequence constituted one complete repetition. The number of completed repetitions in 1 min was recorded.

##### 2.3.1.8 Endurance

The 800/1,000-m run reflects both muscular endurance and the functional levels of the respiratory and cardiovascular systems. The students stood at the starting line and were requested to complete the 800- or 1,000-m test as fast as they could. The time in seconds was recorded.

#### 2.3.2 Questionnaire

The questionnaire included three parts: demographic information of the participants, healthy lifestyle, and PE enjoyment.

##### 2.3.2.1 Demographic information

The first part of the questionnaire collects the participants’ demographic information, including the subjects’ age, gender, grade, major, academic system, ethnicity, place of residence, whether they are the only child in the family, and monthly living expenses.

##### 2.3.2.2 Healthy lifestyle

The second part of the questionnaire gathers participants’ health-related living habits, covering exercise frequency, sleep duration, sedentary time, smoking and alcohol consumption.

Leisure-time exercise frequency among participants was assessed using a single-item scale adapted from prior research (25, 49). Participants were posed the following question: ““Considering a 7-day period (a week), during your leisure time, how often do you engage in any regular activity long enough to work up a sweat (heart beats rapidly)?” Response options were as follows: (1) Never/Rarely (0–1 occasion), (2) Sometimes (2–3 occasions), (3) Often (4 or more occasions).

Participants provided separate reports of their typical bedtime and wake-up time for weekdays and weekends, respectively. We subsequently computed their average daily sleep duration with the formula below: [(Weekday sleep duration × 5) + (Weekend sleep duration × 2)] ÷ 7 (50–52).

Sedentary time was assessed using the Youth Leisuretime Sedentary Behavior Questionnaire (51, 53), which quantifies the daily leisure-time allocation to 12 distinct sedentary behaviors, with separate assessments conducted for weekdays and weekends. For the purposes of this study, sedentary behaviors were categorized into two groups (51): screen time (watching TV/videos + playing computer/video games + Internet surfing) and non-screen sedentary time (doing homework/study without computer + doing homework/study with computer + reading for fun + sitting and talking + talking on the telephone + listening to music + sitting to rest + doing cognitive hobbies + traveling on motorized transport). To address potential over-reporting bias, questionnaire responses were revised to align with actual leisure time, taking into consideration sleep duration, time spent at university, engagement time in physical activities, and the prevalence of each specific behavior prior to the implementation of analytical procedures. The mean daily duration devoted to each sedentary behavior category was computed using the following formula: [(Weekday duration × 5) + (Weekend duration × 2)] ÷ 7.

Current smoking status was evaluated using a single-item question from existing study (50): “How frequently do you smoke cigarettes currently?” The response choices included “I do not smoke,” “less than once a week,” “at least once a week but not daily,” and “every day.” For subsequent analyses, participants who reported smoking at least once a week were categorized as smokers.

The frequency of hazardous alcohol consumption resulting in intoxication was evaluated using a single-item question: “Have you ever consumed such a large quantity of alcohol that you became heavily intoxicated?” The response options were “never,” “once,” “2–3 times,” “4–10 times,” and “more than 10 times.” Drinking frequency exerted a more significant impact on the risk of disease onset than did the quantity of alcohol consumed on each drinking occasion. For example, an analysis of 11,737,467 individuals from the Korean National Health Insurance System database demonstrated that, at equivalent weekly alcohol intake, the risk of gastrointestinal (GI) cancer rose with increasing drinking frequency but declined with greater alcohol consumption per occasion (54). Another study investigating 9,776,956 individuals without atrial fibrillation (AF) found that participants who drank once per week had the lowest risk compared with those who drank twice per week (the reference group). It was concluded that the number of drinking occasions per week was the strongest risk factor for new-onset AF (55). Therefore, past studies typically classified participants who got drunk twice or more per week as hazardous drinkers (50), and this study follows this rule as well.

##### 2.3.2.3 PE enjoyment

The third section of the questionnaire investigates the participants’ enjoyment of PE classes, which was measured using “Factors Influencing Enjoyment of Physical Education” questionnaire (56). This scale comprised 12 items addressing potential factors related to PE classes. Responses were rated on a 5-point Likert scale, where 1 represented Dislike a lot and 5 represented Enjoy a lot. The total score ranged from 12 to 60, with higher scores reflecting a higher degree of enjoyment toward PE lessons. This scale is both valid and adaptable to different languages (22). The internal consistency was also acceptable (Cronbach’s *α* = 0.87) in the present study.

### 2.4 Statistical analysis

The physical fitness data and questionnaire data were matched using student ID numbers. An analytical database was established using Excel software, and the data integrity was checked. Data analysis was conducted using IBM SPSS Statistics version 24.0. A preliminary analysis of the data was performed to verify the fulfillment of the statistical assumptions necessary for the subsequent analyses. The Kolmogorov–Smirnov test indicated that the continuous data followed a normal distribution (*p* > 0.05). Meanwhile, the histogram showed an approximately bell-shaped distribution, and the points in the Q–Q plot were relatively concentrated. Collectively, these results indicate that the continuous variables were normally distributed. Descriptive statistics (numbers and percentages for categorical data, means and standard deviations for continuous data) were used to summarize participants’ questionnaire results. Univariate analysis and Pearson correlation analysis were used to analyze the strengths and directions between the variables. Multiple regression was used to determine the relationship between independent variables (participants’ basic information, health-related lifestyle and enjoyment of PE) and dependent variables (participants’ physical fitness performance). Three models were tested. In the first step (Model 1), control variables (participants’ basic information such as gender, major, academic system, grade, and monthly living expenses) were added, then, in the second step (Model 2), health-related lifestyle factors were added, including exercise frequency, sleep, screen time, non-screen sedentary time, and smoking behavior. Finally, in the third step (Model 3), PE enjoyment was added. No problems of multicollinearity were identified in the models (all tolerance values > 0.948, and all variance inflation factors < 1.037). *p* < 0.05 was considered statistically significant.

## 3 Results

### 3.1 Descriptive statistics of participants’ questionnaire responses

Eventually, 12,840 participants in total (7,116 female and 5,724 male) were eligible for statistical analysis (mean age 19.96 ± 1.12 years), of whom 3,400 (26.48%) were freshmen, 3,222 (25.09%) were sophomores, 3,129 (24.37%) were juniors, and 3,089 (24.06%) were seniors. In terms of majors, the major was clinical medicine, accounting for 79.57%, most of the students were undergraduate-program students (93.59%), with a minority in integrated undergraduate-master (4.10%) or undergraduate-doctoral programs (2.31). 93.94% of participants were Han Chinese, half of them resided in urban areas (50.63%), 60.59% were not the only child, 57.83% had a monthly living cost between 1,000 and 2,000 yuan, 50.03% engaged in extracurricular exercise 2 to 3 times a week, 96.26% did not smoke, 94.01% did not engage in hazardous alcohol consumption, as detailed in Table 2. Moreover, Participants had an average daily sleep duration of 7.10 ± 1.3 h, while their daily screen time and non-screen sedentary time stood at 6.56 ± 3.20 h and 4.56 ± 3.10 h, respectively. Furthermore, their PE class enjoyment score reached 43.92 ± 9.63. These data indicate that the surveyed medical students were evenly distributed in terms of grade and place of birth, and their economic status was at a moderate level (41). More importantly, they exhibited relatively healthy lifestyles, as reflected in the fact that most of them engaged in regular physical activity and had a low prevalence of smoking and risky drinking behaviors (16). On the other hand, they were typically sedentary individuals and spent longer time in sedentary behavior compared with non-medical students (3, 4, 16). Detailed analyses are provided in the Discussion section.

**Table 2:** Descriptive statistics of participants’ questionnaire data (*n* = 12,840).

Qualitative data		Number	Percentage (%)
Gender	Male	5,724	44.58
	Female	7,116	55.42
Grade	Freshman	3,400	26.48
	Sophomore	3,222	25.09
	Junior	3,129	24.37
	Senior	3,089	24.06
Major	Clinical	10,217	79.57
	Non-clinical	2,623	20.43
Academic system	Undergraduate program	12,017	93.59
	8-year integrated undergraduate-master’s program	527	4.10
	9-year integrated undergraduate-doctoral program	296	2.31
Nation	Han Chinese	12,062	93.94
	Ethnic minorities	778	6.06
Place of residence	Urban	6,501	50.63
	Rural	6,339	49.37
Only child	No	7,780	60.59
	Yes	5,060	39.31
Monthly living expenses (yuan)	Below 1,000	388	3.02
	1,001–2000	7,426	57.83
	2001–3,000	4,045	31.50
	3,001–4,000	701	5.46
	Above 4,000	280	2.18
Weekly frequency of exercise	Never/Rarely	3,186	24.81
	Sometimes	6,424	50.03
	Often	3,230	25.16
Current smoking status	No	12,360	96.26
	Yes	480	3.74
Hazardous alcohol consumption	No	12,071	94.01
	Yes	769	5.99

Quantitative data	Mean	Standard deviation
Sleep duration (h)	7.10	1.30
Screen time (h)	6.56	3.20
Non-screen sedentary time (h)	4.56	3.10
PE enjoyment	43.92	9.63

### 3.2 Overall level of physical fitness

The overall mean score of the physical fitness test stood at 70.85 ± 0.33, with scores of 66.25 ± 0.33 for male participants and 75.45 ± 0.34 for female participants. Independent Samples t-test showed that males achieved significantly better results than females in vital capacity, 50-meter sprint and standing-long-jump test, while females performed significantly better in the sit-and-reach test (all *p*s < 0.001, see Table 3 for details). Notably, the overall physical fitness score in the National Student Physical Fitness Test of China represents performance standardized against national assessment criteria, rather than a direct indication of physiological superiority. Since the test sets different standards for male and female participants across individual items, female students achieved higher overall fitness scores, whereas male students outperformed their female counterparts in several raw physical fitness indicators.

**Table 3:** Participants’ performance across various physical fitness test items by gender (mean ± standard errors).

Single index	Physical fitness performance		
	Male	Female	*p*
Height (cm)	176.54 ± 0.08	164.17 ± 0.07	<0.001
Weight (kg)	68.75 ± 0.17	57.09 ± 0.11	<0.001
Body mass index (kg/m^2)	22.02 ± 0.05	21.16 ± 0.04	<0.001
Vital capacity (ml)	4048.30 ± 9.15	2816.38 ± 5.49	<0.001
50-m sprint (s)	7.30 ± 0.01	9.00 ± 0.01	<0.001
Sit-and-reach (cm)	13.84 ± 0.10	18.66 ± 0.07	<0.001
Standing-long-jump (cm)	227.72 ± 0.29	171.15 ± 0.20	<0.001
Pull-ups (counts)	5.13 ± 0.08	/	
Bent-leg sit-up (counts)	/	41.29 ± 0.11	
1,000-m run (s)	250.91 ± 0.43	/	
800-m run (s)	/	239.18 ± 0.31	

### 3.3 Associations between participants’ basic characteristics, healthy lifestyles, PE enjoyment and physical fitness performance

The associations between participants’ basic characteristics, healthy lifestyles, PE enjoyment and physical fitness performance are reported in Tables 4, 5. For participants’ basic characteristics, physical fitness performance was significantly associated with gender (*F* = 4015.43, *p* < 0.001), grade (*F* = 4.42, *p* = 0.004), major (*F* = 194.64, *p* < 0.001), academic system (*F* = 27.06, *p* < 0.001), and monthly living expenses (*F* = 4.88, *p* = 0.001). For categorical data of healthy lifestyles, participants’ physical fitness performance showed significant differences according to weekly frequency of exercise (*F* = 1061.88, *p* < 0.001) and smoking consumption (*F* = 52.04, *p* < 0.001).

**Table 4:** Univariate analysis of the physical fitness score with different characteristics.

Basic characteristics					
	Mean	SE	95% CI	*F*-value	*p*-value
Gender				4015.43	<0.001
Male	66.25	0.33	65.61–66.89		
Female	75.45	0.34	74.78–76.12		
Grade				4.42	0.004
Freshman	70.57	0.35	69.89–71.25		
Sophomore	70.63	0.35	69.95–71.31		
Junior	71.01	0.35	70.32–71.69		
Senior	71.18	0.35	70.50–71.87		
Major				194.64	<0.001
Medical	69.63	0.32	69.01–70.24		
Non-medical	72.07	0.36	71.37–72.38		

**Table 5:** Correlation matrix of healthy lifestyles, PE enjoyment and physical fitness performance.

Variables	1	2	3	4	5
1. Sleep duration	–				
2. Screen time	−0.00	–			
3. Non-screen sedentary time	−0.04**	0.07**	–		
4. PE enjoyment	−0.00	−0.06**	−0.04**	–	
5. Physical fitness performance	0.05**	−0.26**	−0.30**	0.21**	–

**p < 0.01.

For continuous variables, sleep duration and PE enjoyment were positively associated with physical fitness performance, whereas sedentary behaviors (screen time and non-screen sedentary time) exhibited a negative association with physical fitness performance. Moreover, non-screen sedentary time was positively correlated with screen time, whereas it was negatively correlated with sleep duration. Furthermore, PE enjoyment was negatively associated with both screen time and non-screen sedentary time. Additionally, no statistically significant associations were observed among the other continuous variables (see Table 5).

### 3.4 Regression analysis of the influencing variables of physical fitness

Factors that were statistically significant in the univariate or Pearson analysis were used as independent variables, while the total score of participants’ physical fitness was used as the dependent variable. Hierarchical multiple regression analysis was performed to measure these variables, and the variable assignments are shown in Table 6. Variables such as grade, academic system, monthly living expenses, and exercise frequency were treated as ordinal predictors. The variable coding strategy adopted in this study was derived from existing literature (57). Table 7 presents the associations of healthy lifestyles and PE enjoyment with physical fitness (Supplementary Table 1). In Model 1, gender, major, academic system, grade and monthly living expenses were added to the model as control variables, and they were found to be significant predictors of physical fitness performance. Grade was a positive predictor of physical fitness performance (*β* = 0.03), while monthly living expenses (*β* = −0.02) and academic system (*β* = −0.06) negatively predicted physical fitness. Female students (*β* = 0.48) were more likely to have better physical fitness scores, while medical students (*β* = −0.09) were more likely to have worse physical fitness performance. Healthy lifestyles were added to the second model. Exercise frequency (*β* = 0.29), sleep duration (*β* = 0.03) and non-screen sedentary time (*β* = 0.02) positively predicted physical fitness, whereas screen time (*β* = −0.22) and smoking (*β* = −0.25) were negative predictors of physical fitness. The final models included PE enjoyment. PE enjoyment significantly predicted physical fitness (*β* = 0.15). After controlling for PE enjoyment, the negative correlation between smoking and physical fitness became non-significant (*p* = 0.15).

**Table 6:** Coding methods for independent variables in the analysis.

Independent variable		Assignment method
Basic characteristics	Gender	0 = Male, 1 = Female
	Grade	1 = Freshman, 2 = Sophomore, 3 = Junior, 4 = Senior
	Major	0 = non-medical, 1 = medical
	Academic system	1 = Undergraduate program, 2 = 8-year integrated bachelor’s-master’s program, 3 = 9-year integrated bachelor’s – doctoral program
	Monthly living expenses (RMB)	1 = Below 1,000, 2 = 1,001–2000, 3 = 2001–3,000, 4 = 3,001–4,000, 5 = above 4,000
Healthy lifestyles	Exercise frequency	1 = 0 to 1 time, 2 = 2–3 times, 3 = 4 times or more
	Smoking	0 = No, 1 = Yes
	Sleep duration	Continuous (original scores)
	Screen time	Continuous (original scores)
	Non-screen sedentary time	Continuous (original scores)
PE Enjoyment		Continuous (original scores)

**Table 7:** The associations between healthy lifestyles, PE enjoyment and physical fitness performance.

Variables	Model 1		Model 2		Model 3	
	*β*	*p*	*β*	*p*	*β*	*p*
Gender (Ref: male)	0.48	<0.001	0.45	<0.001	0.45	<0.001
Major (Ref: non-medical)	−0.09	<0.001	−0.10	<0.001	−0.09	<0.001
Academic system (Ref: Undergraduate program)	−0.06	<0.001	−0.05	<0.001	−0.05	<0.001
Grade	0.03	<0.001	0.02	0.001	0.02	<0.01
Monthly living expenses (Ref: less than 1,000 yuan)	−0.02	0.03	−0.01	0.11	−0.01	<0.05
Exercise frequency	*/*	/	0.29	<0.001	0.28	<0.001
Screen time	*/*	/	−0.22	<0.001	−0.22	<0.001
Sleep	*/*	/	0.03	<0.001	0.03	<0.001
Non-screen sedentary time	*/*	/	0.02	0.03	0.02	<0.01
Smoke	*/*	/	−0.02	0.03	−0.01	0.15
PE Enjoyment	*/*	/	/	*/*	0.15	<0.001
*R^2^*	0.24		0.39		0.42	
*∆R^2^*	0.24		0.16		0.02	
*F*-change	796.76		660.25		505.86	
*p*	< 0.001		< 0.001		< 0.001	

Values are standardized linear regression coefficients (β). All tests in the table were subjected to the Benjamini and Hochberg correction for multiple comparisons.

**Figure 1:**
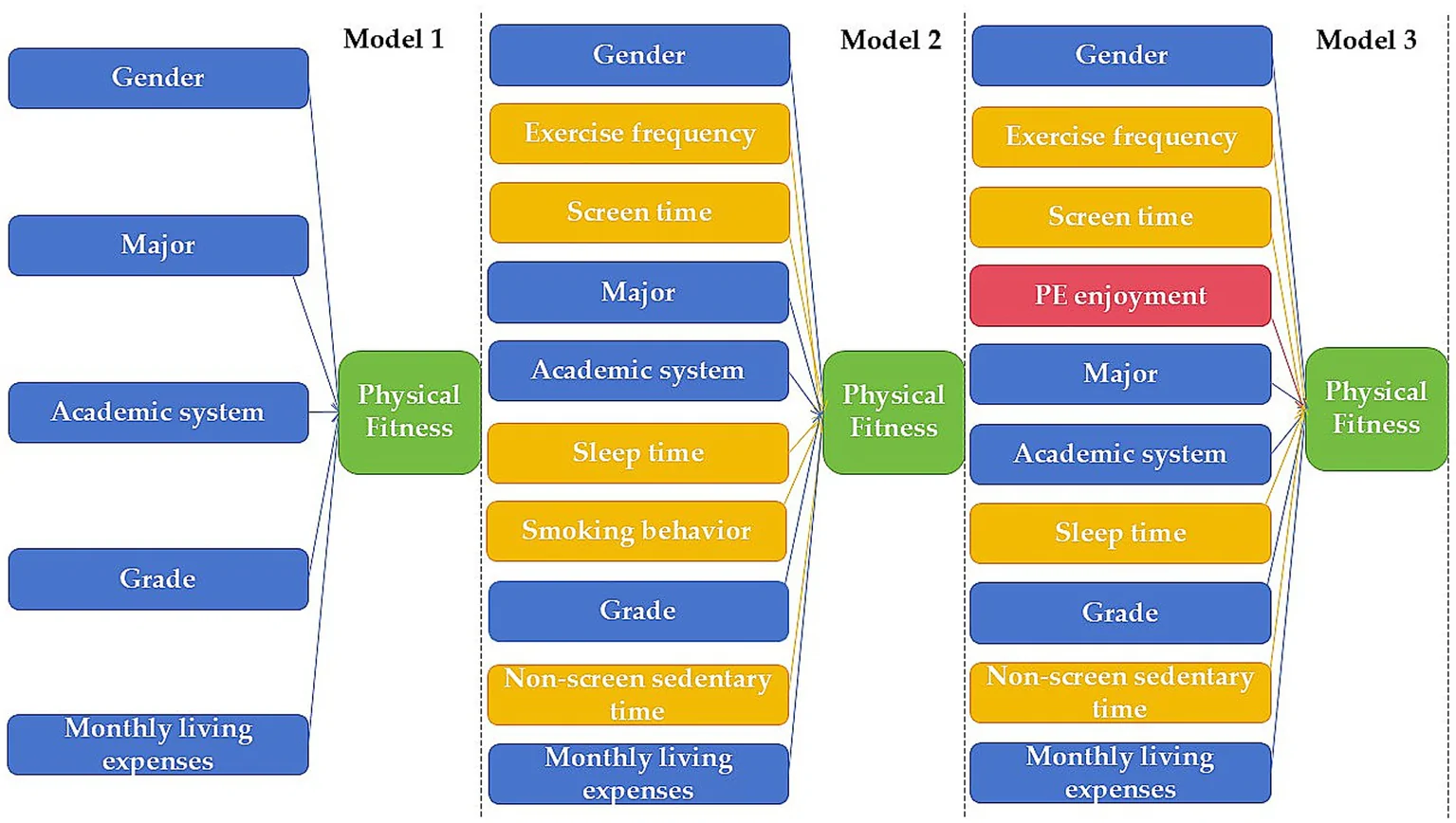
The three tested models (Left: Model 1; Middle: Model 2; Right: Model 3). Blue represents control variables, orange represents health-related lifestyle factors, and red represents PE enjoyment.

Moreover, the associations between demographic variables and physical fitness ranked in descending order was: gender, major, academic system, grade, and monthly living expenses. After adding factors related to healthy lifestyle habits, the factors are ranked in descending order of their correlation with physical fitness as follows: gender, exercise frequency, smoking behavior, screen time, major, academic system, sleep time, grade, non-screen sedentary time, and monthly living expenses. Following the inclusion of PE enjoyment, the association between smoking behavior and physical fitness diminished substantially. At this stage, the factors associated with physical fitness are ranked hierarchically as follows: gender, exercise frequency, screen time, PE enjoyment, major, academic system, sleep time, grade, non-screen sedentary time, and monthly living expenses (Figure 1).

Hierarchical multiple regression analysis revealed that Model 1, which incorporated control variables (gender, major, academic system, grade, and monthly living expenses), explained 24% of the variance in physical fitness. With the addition of factors related to healthy lifestyle habits, Model 2 increased the explained variance in physical fitness by a further 16%. Subsequently, the inclusion of PE enjoyment in Model 3 contributed an additional 2% of explained variance. In short, from Model 1 to Model 3, the explained variance rose from 24 to 42%.

## 4 Discussion

This study reveals the overall level of physical fitness tests and explains the associations of physical education enjoyment and healthy lifestyle with physical fitness in a large sample of medical students. Our findings indicate that although relatively uncontrollable factors (e.g., gender, major, academic system, grade, and monthly living expenses) show significant correlations with physical fitness, relatively self-controllable factors (healthy lifestyle habits) and interactions with the external environment (PE enjoyment) also exhibit strong associations with physical fitness. This suggests that subjective initiative and PE interventions may correlate positively with physical health.

### 4.1 Core findings

First, the physical fitness of students in medical universities was found to be at a medium level, which is significantly below the target for the excellent and good rate of college students’ physical fitness stipulated in the Healthy China 2030 Initiative. With regard to specific indicators, participants’ BMI was significantly higher than that of medical students in previous years, consistent with the widespread rise in obesity prevalence among Chinese college students following the COVID-19 pandemic (48). No significant changes were observed in the 50-m sprint, sit-and-reach, or standing long jump compared with medical students in prior years (58, 59). For male students, pull-up and endurance run performance showed some improvement compared with 3 years ago (59), but remained below the level observed 10 years ago (58). For female students, bent-leg sit-up performance improved compared with female medical students in previous years, and endurance run performance was similar to that of female medical students in prior years (59), but both were lower than the overall average of female college students in previous years (3). Moreover, vital capacity of all participated students decreased markedly relative to medical students in previous years (59), which, together with elevated BMI, contributed to the overall decline in participants’ physical fitness (37). Notably, due to differences in population, region, school level, measurement conditions and other factors among the subjects involved in the above studies, whether the downward trend in the physical fitness of medical students has been reversed remains to be further explored in future research.

In terms of total scores, female medical students seemed exhibit higher physical health scores than male students, which is consistent with previous findings and related to the significant gender differences in obesity rates. A physical fitness test conducted among 2,291 medical students in Shenyang, China, in May 2019 (before the COVID-19 pandemic) revealed that the obesity rate of female students was significantly lower than that of male students, and their physical fitness level was significantly better (59). A statistical analysis of physical fitness test scores among 103,072 Chinese college students from 2019 to 2021 (after the pandemic) showed that the overweight and obesity rate among male students increased continuously year by year (from 22.53% in 2019 to 29.25% in 2021), and their overall physical fitness level was also significantly lower than that of female students (48). A physical fitness survey conducted 10 years ago among 8,548 medical students and 3,060 non-medical college students in China found that the obesity rate of male students was significantly higher than that of female students, and obesity was strongly associated with poorer physical fitness performance (60), especially in the standing long jump and endurance running (61). Collectively, these results indicate a significant gender gap in physical fitness among Chinese medical students, and the upward trend in the obesity rate among male medical students has not been effectively mitigated.

Meanwhile, the study indicated that students with higher monthly living expenses exhibited poorer physical fitness, which may be associated with an increased prevalence of obesity resulting from greater food intake (62). Other research has suggested that socioeconomic status exhibits an inverted U-shaped relationship with health literacy, such that both excessively high and low socioeconomic levels contribute to inferior health beliefs and behaviors (41). Some scholars have argued that students from lower socioeconomic backgrounds tend to have poorer physical fitness due to a lack of resources such as facilities (63). However, most of these studies focused on children or adolescents rather than exclusively on university students. The participants in the present study were all medical college students from coastal regions, where institutions are equipped with abundant and free sports facilities and inexpensive, nutritious canteens. These conditions mitigate the potential negative association between low socioeconomic status and physical fitness. This also explains why no significant difference in physical fitness was observed across birthplaces in the study population, as participants from all regions could access the same shared health resources on campus.

Moreover, non-clinical students exhibited better physical fitness than clinical students, which may be related to the longer schooling duration of clinical majors compared with non-clinical majors. This also explains the finding of the present study that students in the integrated undergraduate-master’s program or the integrated undergraduate-doctoral program had worse physical fitness than those in the standard four- or five-year undergraduate programs. In medical colleges and universities, there are direct bachelor-master or bachelor-doctorate programs, which allow students to progress directly from undergraduate medical studies to master’s or doctoral programs and complete their studies within 8 to 9 years (64). Accordingly, the academic requirements for these students are stricter than those for regular undergraduate medical students, they must achieve outstanding academic performance during the undergraduate stage to successfully advance to the master’s or doctoral phase. A follow-up study of physical fitness data among 634 medical students who graduated in 2022 revealed a downward trend in their physical fitness year by year (37).

More importantly, this study reveals that medical students are a highly sedentary group, with a daily sedentary time of 11.3 h, approximately 2 h longer than that of non-medical students (3, 4). In particular, their daily screen time is approximately 3 h more than that of non-medical students (16). Previous studies have confirmed a negative correlation between sedentary behavior and physical fitness (65), especially when sedentary time consists of screen time (15, 66). Meanwhile, sedentary time also negatively affects exercise duration and frequency. A survey of 21,612 Spanish university students found that non-physical activity time was strongly associated with sedentary time (16). Furthermore, numerous studies have demonstrated that sedentary behavior negatively impacts sleep quality (67–69), whereas appropriate physical activity improves sleep quality (14, 17). Therefore, sedentary behavior exerts adverse effects on physical fitness by reducing physical activity and impairing sleep quality. Therefore, the present study has shown that medical students sleep an average of 1 h less per day than students in other majors (4). These findings also strongly support the results of the present study, namely that exercise frequency and sleep duration positively predict the physical fitness of medical students, while screen-based sedentary time negatively predicts their physical fitness.

Notably, not all sedentary behaviors have negative links to physical fitness. Due to heavy academic demands, medical students spend most of their time studying, resulting in relatively long non-screen sedentary time. In this study, non-screen sedentary time included activities such as doing homework and studying, reading, cognitive hobbies, socializing, resting, and transportation. In contrast to screen time, which mainly consisted of TV viewing, screen-based games, and Internet surfing, non-screen sedentary time additionally involved non-leisure study and cognition-enhancing activities. This may account for the opposite correlations of screen time and non-screen sedentary time with physical fitness observed in the present study. A survey of 1,296 German students found that only weekend sedentary time is negatively associated with health, indicating that only leisure sedentary time has a detrimental effect on health (70). Thus, the non-screen sedentary time of medical students is devoted to medical learning rather than leisure activities. Meanwhile, studying medicine helps cultivate better health awareness (41), which also explains why non-screen sedentary time was found to positively predict the physical fitness of medical students in this study. Studies distinguishing the differential effects of various sedentary behaviors on physical fitness have also reported that only screen-based sedentary time, rather than non-screen-based sedentary time, negatively impacts physical fitness (71). Although one research has suggested that non-screen sedentary time and screen time both negatively affect physical fitness (12), the participants in that study were children (mean age 9.1 years) and adolescents (mean age 13.6 years), much younger than the medical students in the present study (mean age 20.0 years). It is worth noting that our interpretation linking non-screen sedentary time to study-related and cognitively advantageous activities among medical students is tentative. Alternative explanations, namely residual confounding, measurement bias, suppression effects and disparities in time management, warrant consideration. For this reason, we are unable to draw the simplistic conclusion that increased non-screen sedentary time improves physical fitness. In fact, our results confirm a negative correlation between sedentary behavior and physical fitness. It is hoped that the findings of this study, particularly those regarding sedentary time, can inform medical students that given their inherent sedentary lifestyle driven by academic training, they should minimize sedentary time—especially screen-based sedentary time—and engage in more physical activity to develop sound health habits and maintain good physical fitness.

Results regarding PE enjoyment indicate that the participating medical students experienced substantial enjoyment in PE classes, which may be associated with their fulfillment of autonomy, competence, and relatedness during these classes. Providing students with opportunities to experience autonomy, competence, and relatedness in PE plays a critical role in promoting learning satisfaction and optimizing learning outcomes (72, 73). Studies have found that students who perceive a high level of autonomy in PE classes exhibit positive classroom behaviors (e.g., greater engagement in class activities) (74) and demonstrate strong intentions to engage in physical activity during leisure time (73) as well as actual physical activity behaviors (72). Synthesizing existing research, adjusting teaching strategies to prioritize students’ autonomy and active classroom participation may correlate with superior physical education learning outcomes, higher classroom engagement, and more spontaneous physical activity behaviors. Accordingly, targeted institutional interventions may be implemented to improve medical students’ physical health. For instance, greater autonomy should be provided to students in PE curriculum design, appropriate goals should be set progressively so that students can achieve them through effort and thus develop a sense of competence, and a positive learning environment should be established between instructors and students as well as among peers.

Previous studies have demonstrated that smoking is harmful to physical health. Based on the regression results, the present study found that when PE enjoyment was incorporated, the negative correlation between smoking and physical fitness was no longer significant. Zhang et al. (75) found that participation in physical education classes was negatively correlated with unhealthy lifestyle behaviors such as smoking. Smoking has adverse effects on cardiopulmonary function, cardiac autonomic nervous function, and vascular function (76–78), whereas physical activity exerts promoting effects on these functions (79). Meanwhile, the results showed that only smokers had significantly lower physical fitness than non-smokers, whereas no significant difference was observed between drinkers and non-drinkers. These results are supported by previous studies (8, 80). Carballo-Fazanes (16) surveyed 608 university students in Spain and found that only smoking, rather than alcohol consumption, was significantly negatively correlated with physical activity, although the proportion of students who engaged in regular exercise was higher among drinkers than among non-drinkers. Other studies have also found that non-smokers have better health literacy (41) and better body appreciation (50) than smokers.

Among the demographic predictors, gender exhibited the strongest association with physical fitness, representing a practically meaningful difference in overall fitness performance between male and female students under the standardized national fitness evaluation system. By comparison, the significant but extremely small coefficients of grade and monthly living expenses indicate that although these variables reached statistical significance, their practical contribution to fitness variation is negligible in the real-world student population. Similarly, academic system and student major demonstrated weak predictive effects, suggesting that demographic factors play a relatively limited role in shaping physical fitness compared with behavioral factors in this sample. In terms of healthy lifestyle indicators, exercise frequency emerged as the dominant positive behavioral predictor and remained robust after further controlling for PE enjoyment, confirming its substantial practical value in promoting student physical fitness. In contrast, although sleep duration and non-screen sedentary time showed statistically positive associations with fitness, their near-zero effect sizes indicate minimal practical significance. These trivial associations suggest that slight variations in daily sleep length and low-intensity non-screen sedentary behavior are insufficient to produce meaningful differences in overall physical fitness among college students. Meanwhile, screen time and smoking behavior demonstrated considerable negative correlations with physical fitness, highlighting unhealthy behavioral patterns as critical risk factors for declining student physical health. Notably, after incorporating PE enjoyment into the full model, the previously significant association between smoking and physical fitness became statistically non-significant and substantially diminished in magnitude. This change indicates that PE enjoyment serves as an important confounding and contextual factor, which might partially offset the negative fitness impact of smoking in the student population. In the final hierarchical model, the relative importance of predictors was clearly reordered: gender, exercise frequency, screen time, and PE enjoyment constituted the core practically meaningful determinants of physical fitness, whereas sleep duration, non-screen sedentary time, and economic variables exerted only marginal and trivial influences. Collectively, these findings highlight that in large-sample epidemiological fitness research, statistical significance alone cannot represent practical value. This study further confirms that voluntary exercise participation, reduced screen exposure, and positive PE affective experience are the most actionable, modifiable, and practically significant targets for university physical health intervention, while minor variations in sleep and non-screen sedentary behavior have limited applied value for fitness promotion strategies.

Finally, this study found that relatively self-controllable factors (healthy lifestyle habits) and interactions with the external environment (PE enjoyment) were significantly correlated with physical fitness, suggesting the positive link between medical students’ own subjective initiative and effective school-based interventions on physical fitness. Grounded in self-determination theory, internalized motivation is more conducive to the achievement of health behaviors (81). The participants in this study were all medical students, whose health knowledge and awareness may facilitate the development of healthy lifestyle habits (39–41). This also explains the finding that only 3.9% of medical students were regular smokers and 6.0% were drinkers, a prevalence lower than that among college students from non-medical universities (16). Furthermore, numerous studies have shown that enjoyment in physical education is negatively correlated with BMI (27), and positively correlated with physical education engagement (23), physical literacy (82), and moderate-to-vigorous physical activity intensity during physical education (27). Therefore, simply emphasizing skill training is insufficient, diversifying PE content, enhancing fun and experiential value, and responding to students’ psychological needs can effectively promote exercise motivation and habit formation. In addition, both this study and existing studies (3, 12, 17) indicate that physical exercise can alleviate the negative effects of sedentary behavior on physical fitness. This result also explains the finding of the present study that medical students’ physical fitness improves with grade level, which could be attributed to the intervention effect of university physical education courses. Notably, this moderating effect is influenced by gender. Studies have shown that among female students, physical activity improves physical fitness mainly by reducing sedentary behavior, whereas among male students, it enhances physical fitness by reducing obesity and improving body composition (4, 51, 83). Accordingly, when designing physical education curricula and delivering instruction, universities and teachers should adjust course content, key points, and difficulties according to the gender, characteristics, and needs of students. This is also crucial for increasing students’ enjoyment in physical education and promoting their physical fitness.

### 4.2 Strengths, limitations and future directions

The present study demonstrated that healthy lifestyles and PE enjoyment were significantly and positively associated with physical fitness among Chinese medical students. To our knowledge, this is the first study to systematically explore the combined associations between these two domains and physical fitness among this population. The findings of this study suggest that the physical fitness of medical students is associated with many factors. Universities and teachers should pay close attention to the physical fitness of medical students, promote healthy lifestyle and carry out targeted health education for them. In daily life, strengthen the promotion of physical health and equip with comprehensive health facilities, so as to enrich students’ after-school life and reduce their sedentary behavior, especially screen time. Regarding the curriculum design of physical education, in addition to the necessary instruction of sports skills, teaching content should be diversified to enhance the enjoyment and experience of physical activity, foster students’ interest in exercise, help them form regular exercise habits, and continuously improve the physical fitness of medical students. Based on the application of self-determination theory in the field of education (84, 85), teachers can achieve this goal by providing students with opportunities to gain autonomy, competence, and relatedness.

The limitations of this study and implications for future research are as follows. Firstly, the sample could not truly represent the entire medical students in China. Although participants in this study came from various regions across the country, they all studied at medical colleges in Jiangsu Province. As a relatively developed province in China, Jiangsu has sufficient teaching staff and sports facilities in schools. Therefore, the results of this study (especially regarding the relationship between monthly living expenses and physical fitness) cannot be directly generalized to other provinces. In other words, the findings regarding living expenses, facility access, and lifestyle behaviors may not apply to students in less-resourced settings. In addition, the distribution of participants across universities and campuses was not evaluated. Given that students are clustered within institutional environments, school- or campus-level confounding effects may exist. The inability to conduct multilevel modeling or cluster-adjusted analyses represents a key limitation of the present study. However, the large sample size is one of the greatest strengths of this study. Future studies will attempt to cover more regions and incorporate the distribution of participants across different universities and campuses to improve generalizability and provide a more comprehensive investigation into the overall physical fitness level of Chinese medical students. Additionally, the current study was a cross-sectional study, therefore, there is a risk of overinterpretation in the practical recommendations proposed herein. We could not establish a cause-and-effect relation but only identified association between PE enjoyment, healthy lifestyle and physical fitness index. Future research should employ multicenter designs encompassing regions with varying levels of policy support and curriculum development, and longitudinal follow-up to better identify causal pathways and key determinants of medical students’ physical fitness. Thirdly, although the selected measures have been widely used in previous studies and demonstrate good reliability and validity, the use of single-item measures for key variables such as exercise frequency, smoking, and alcohol consumption may limit measurement accuracy. Given the large sample size (13,562 participants) and the extensive scope of the survey (covering physical fitness level, demographic information, lifestyle, and PE enjoyment), we adopted single-item indicators for certain content domains. This decision was made to avoid fatigue effects among participants resulting from an excessive number of test items and to control the overall testing duration (ensuring all tasks were completed within approximately 90 min per participant). Moreover, the physical activity questionnaire item captures both the frequency and moderate intensity of exercise. Participants were asked how often they took part in regular activity lasting long enough to induce sweating and a rapid heartbeat. Notably, not all participants are familiar with the definition of moderate-intensity physical activity, nor do they typically monitor exercise intensity during activity. Accordingly, this descriptive question helps standardize respondents’ understanding of moderate-intensity exercise, thereby yielding more objective and reliable data. Admittedly, including the duration of such activity would strengthen the measurement. Therefore, future research will attempt to adopt multi-item and multi-dimensional measurement methods to obtain more comprehensive and accurate information from participants. Finally, some lifestyle variables were self-reported, which may introduce recall bias or social desirability bias. To reduce social desirability bias, we stated in the informed consent that participants’ personal data would be confidential and only used for academic purposes. Additionally, entries with apparent errors, such as combined sedentary and sleep time exceeding 24 h, were removed. In the future, monitoring systems such as electronic watches and AI-based automatic devices may be adopted to collect data on students’ physical activity, sleep and sedentary behavior, so as to obtain more objective, comprehensive and reliable results.

## 5 Conclusion

This study demonstrates that due to the unique nature of medical education (i.e., a longer curriculum and higher cognitive demands), medical students constitute a highly sedentary population with moderate levels of physical fitness. Healthy lifestyles characterized by sufficient physical activity, appropriate non-screen sedentary time, and adequate sleep, together with higher levels of physical education enjoyment, are positively correlated with physical fitness of medical students. In contrast, prolonged screen behavior and smoking were identified as major risk factors for poor physical fitness. Furthermore, once PE enjoyment was taken into account, the negative correlation between smoking and physical fitness lost significance. These results indicate that cultivating healthy lifestyles via individual intrinsic initiative and elevating PE quality may contribute to better physical fitness among medical students. Medical institutions and educators should strengthen health education, provide targeted interventions to foster healthy lifestyle habits among medical students, and enhance enjoyment in physical education (PE) classes. Such efforts may not only help improve the physical fitness of the medical student population and provide sustained impetus for the future development of the healthcare sector, but could also represent a critical step in implementing the Healthy China 2030 Initiative and advancing China’s policy of building a leading sports nation.

## Data Availability

The data that support the findings of this study are available from the corresponding author upon reasonable request.

## Footnotes

1
https://www.wjx.cn/
