# Supplementary material for: Associations of healthy lifestyles and physical education enjoyment with physical fitness among Chinese medical students: a cross-sectional study
Source: Front Public Health. 2026 Aug 26;14:1894015. doi: 10.3389/fpubh.2026.1894015 (PMC13553788; doi:10.3389/fpubh.2026.1894015)
Supplement: Supplementary file 1 [file Table_1.docx]

**Supplementary Table 1. The associations between healthy lifestyles, PE enjoyment and physical fitness performance.**

| **Variables** | **Model 1** | | **Model 2** | | **Model 3** | |
| --- | --- | --- | --- | --- | --- | --- |
|  | ***B*** | **95%CI** | ***B*** | **95%CI** | ***B*** | **95%CI** |
| Gender (Ref: male) | 9.38 | 9.08 ~ 9.67 | 8.92 | 8.65 ~ 9.19 | 8.94 | 8.67 ~ 9.20 |
| Major (Ref: non-medical) | -2.26 | -2.63 ~ -1.89 | -2.38 | -2.71 ~ -2.05 | -2.26 | -2.59 ~ -1.94 |
| Academic system (Ref: Undergraduate program) | -1.62 | -2.04 ~ -1.20 | -1.39 | -1.96 ~ -1.01 | -1.32 | -1.69 ~ -0.95 |
| Grade | 0.27 | 0.14 ~ 0.40 | 0.21 | 0.09 ~ 0.33 | 0.18 | 0.06 ~ 0.29 |
| Monthly living expenses (Ref: less than 1000 yuan) | -0.23 | -0.43 ~ -0.03 | -0.14 | -0.32 ~ 0.04 | -0.18 | -0.35 ~ -0.00 |
| Exercise frequency | */* | / | 4.06 | 3.87 ~ 4.25 | 3.81 | 3.62 ~ 4.00 |
| Screen time | */* | / | -0.69 | -0.74 ~ -0.69 | -0.67 | -0.72 ~ -0.63 |
| Sleep | */* | / | 0.24 | 0.14 ~ 0.34 | 0.25 | 0.15 ~ 0.35 |
| Non-screen sedentary time | */* | / | 0.05 | 0.01 ~ 0.09 | 0.06 | 0.02 ~ 0.11 |
| Smoke | */* | / | -0.83 | -1.55 ~ -0.11 | -0.52 | -1.23 ~ 0.19 |
| PE Enjoyment | */* | / | / | */* | 0.16 | 0.14 ~ 0.17 |

Values are unstandardized linear regression coefficients (B) and 95% confidence intervals for the unstandardized regression coefficients.
